# High Ki67, Bax, and thymidylate synthase expression well correlates with response to chemoradiation therapy in locally advanced rectal cancers: proposal of a logistic model for prediction

**DOI:** 10.1038/sj.bjc.6605105

**Published:** 2009-06-02

**Authors:** M Kikuchi, T Mikami, T Sato, W Tokuyama, K Araki, M Watanabe, K Saigenji, I Okayasu

**Affiliations:** 1 Department of Pathology, Kitasato University School of Medicine, Sagamihara, Kanagawa, Japan; 2 Department of Gastroenterology, Kitasato University School of Medicine, Sagamihara, Kanagawa, Japan; 3 Department of Surgery, Kitasato University School of Medicine, Sagamihara, Kanagawa, Japan

**Keywords:** Ki67, Bax, thymidylate synthase, chemoradiation, rectal cancer

## Abstract

**Background:**

Recently, preoperative chemoradiation therapy (CRT) for rectal cancer has been increasingly used as a neoadjuvant treatment. In the present study, the relation between histological response to CRT and immunohistochemical markers in biopsy specimens was investigated.

**Methods:**

Biopsy specimens from a total of 60 patients were collected before preoperative CRT with S-1 and irinotecan, and liniac 45 Gy. Immunohistochemical staining for Ki67, Mcm3, Bax, Bcl-2, ssDNA, Grp78, thymidylate synthase (TS), dihydropyrimidine dehydrogenase (DPD), CD34, vascular endothelial growth factor, nestin, and L-type amino-acid transporter 1 was performed to allow comparison of the Ki67 labelling index (LI), Bax score, TS score, DPD score, microvessel density by CD34, and Grp78 score with cancer regression.

**Results:**

When the cases were divided into responders (Dworak grades 3 and 4) and non-responders (grades 1 and 2) groups, good correlations were evident with Ki67 LI, Bax, Grp78, and TS expression. On multiple logistic regression analysis, Ki67 LI, Bax, and TS scores were found to be independent factors. With their use in a logistic model, *P*-values could predict responder cases with a sensitivity of 82.8% and a specificity of 83.9%.

**Conclusion:**

Using this system, treatment strategy for locally advanced rectal cancers can be determined before chemoradiation.

Chemoradiation therapy (CRT) is increasingly used in neoadjuvant approaches for rectal cancer. Although in locally advanced cases there is generally no improvement of overall survival, local control is better than with postoperative CRT (Sauer *et al*, 2004). Combining irinotecan with 5-fluorouracil (5-FU) and leucovorin (LV) chemotherapy can provide higher rates of tumour regression, progression-free survival, and overall survival for metastatic colorectal cancer (Saltz *et al*, 2000). Recently, our group has developed a novel protocol for neoadjuvant CRT combining S-1 with irinotecan and radiation, allowing a complete pathological response rate of 31.6% to be achieved (Sato *et al*, 2007). It is well established that a pathological complete response (CR) or near CR (>95% pathological response) is significantly linked with improved patient survival (Ruo *et al*, 2002; Guillem *et al*, 2005).

S-1 is a novel oral fluoropyrimidine, combining tegafur (FT), 5-chloro-2,4-dihydroxypyridine (gimeracil or CDHP), and potassium oxonate (oteracil potassium or Oxo). FT is a prodrug for 5-FU that acts as an effector. CDHP reversibly inhibits the degradation of 5-FU by dihydropyrimidine dehydrogenase (DPD), resulting in prolonged high concentrations of 5-FU in the blood (Sato *et al*, 2007). Irinotecan (CPT-11) inactivates topoisomerase I through the formation of stable topoisomerase I–DNA cleavable complexes (Hsiang *et al*, 1985; Hsiang and Liu, 1988; Hertzberg *et al*, 1989). Interaction of the trapped cleavable complex with a replication fork results in replication arrest and fork breakage, finally leading to cell death (D'Arpa and Liu, 1989).

High proliferative activity examined with Ki67 and proliferating cell nuclear antigen (PCNA) staining (Willett *et al*, 1995), high Bax expression (Chang *et al*, 2005), and high thymidylate synthase (TS) (Negri *et al*, 2008) have been demonstrated to predict regression. However, these factors have been treated as univariate factors. No clinically applicable system for prediction of response of CRT has been proposed. We therefore have investigated cell proliferation, apoptosis, apoptosis-associated protein, expression of glucose-regulated protein 78 (Grp78), TS, DPD, and angiogenesis in biopsy samples in an attempt to develop a predictive system. Selection of these parameters was for the following reasons.

Thymidylate synthase provides *de novo* thymidylate for DNA synthesis, catalysing the methylation of deoxyuridine monophosphate to deoxythymidine monophosphate (Danenberg, 1977). The activity of 5-FU mainly depends on intracellular conversion to its active metabolite, 5-fluoro-2′-deoxyuridine-5′-monophosphate, which inhibits DNA synthesis by forming a stable complex with TS in presence of folates (Pinedo and Peters, 1988), and then initiates cell-cycle arrest or cell death. In general, high expression of thymidine phosphorylase and low expression of DPD in tumours are considered to result in higher intratumoural concentration of 5-FU (Jakob *et al*, 2005).

Glucose-regulated protein 78 is a member of the Hsp70 superfamily of heat-shock proteins whose increased expression is part of a coordinated protein response required to alleviate endoplasmic reticulum stress (Misra *et al*, 2005), maintain endoplasmic reticulum function, and protect cells against cell death. Glucose-regulated protein 78 may confer resistance against adriamycin- and etoposide-mediated apoptosis in cancer cells through inhibition of Bax and caspase-7 activation (Reddy *et al*, 2003; Davidson *et al*, 2005; Ermakova *et al*, 2006; Lee *et al*, 2006; Ranganathan *et al*, 2006).

Recently, the concept of cancer stem cells has attracted increasing attention with regard to human cancers (Wang and Dick, 2005). Minichromosome maintenance (Mcm) proteins 2–7 are present through all phases of the proliferative cell cycle, but are absent in ‘out-of-cycle’ states, suggesting functions as replication licensing factors (Stoeber *et al*, 2001), and Dudderidge *et al* (2005) have proposed that the Mcm2-Ki67 labelling index (LI) reflects the presence of non-proliferating dormant ‘cancer stem’ cells, associated with reduced disease-free survival in renal cell carcinoma cases.

It was reported that high intratumoural microvessel density (MVD) and vascular endothelial growth factor (VEGF) were correlated with poor prognosis of colorectal cancer (Des Guetz *et al*, 2006). It has been also reported that VEGF-positive rectal cancer was resistant to radiotherapy (Zlobec *et al*, 2008). Nestin, a class VI intermediate filament protein, has recently received attention as a marker for detecting newly formed endothelial cells (Teranishi *et al*, 2007).

As for apoptosis, cancer cell apoptosis in biopsy before CRT was correlated with tumour regression whereas apoptosis inhibitory protein Bcl-2 expression indicated no correlation with regression (Rödel *et al*, 2002). In addition, L-type amino-acid transporter 1 (LAT1) is highly expressed in malignant tumours to support growth and proliferation, and the inhibition of LAT1 activity led to cancer cell apoptosis (Kim *et al*, 2008).

Using these parameters, multiple logistic regression analysis was adopted to generate a model for predicting response to preoperative CRT.

## Materials and methods

A total of 60 cases of rectal cancer treated with preoperative CRT were collected. The patients' clinical criteria were previously reported (Sato *et al*, 2007). Briefly, all had previously untreated locally advanced distal rectal cancer T3 or T4, N0-2, and M0 (UICC classification) (Sobin and Wittekind, 2002), with an Eastern Cooperative Oncology Group performance status of 0-2 (Oken *et al*, 1982). Ages were 20–80 years at enrolment.

Biopsy materials of the 60 cases were endoscopically obtained from the rectal cancers before the initiation of therapy, at least two pieces of carcinoma being sampled for each case. The histological typing was in accordance with the WHO classification (Hamilton and Aaltonen, 2000). Tumour size was measured using double-contrast barium enema in 56 cases, but no X-ray photographs were available for 4 cases. Clinical tumour node metastasis (TNM) stage was judged with computed tomographic scans and/or magnetic resonance images. Because images were not available for two cases, clinical TNM stage could not be determined for these. A summary of clinical data of the cases is shown in Table 1. All patients received preoperative chemoradiation as follows: radiotherapy was administered in liniac fractions of 1.8 Gy per day, 5 days per week. The total dose of radiation was 45 Gy. S-1 (80 mg m^−2^ per day) and was given orally after breakfast and dinner on days 1–5, 8–12, 22–26, and 29–33. Irinotecan (80 mg m^−2^) was administered as a continuous i.v. infusion for 90 min on days 0, 8, 22, and 29. Radical surgery was performed at least 4–6 weeks after the completion of 5 weeks of chemoradiation. The dose of S-1 was in accordance with the manufacturer's guideline (Taiho Pharmaceuticals Co. Ltd, Tokyo, Japan). The recommended dose of irinotecan was examined in our previous study (Sato *et al*, 2007). This protocol was started in 2004 with approval of the ethics committee of Kitasato University Hospital. All patients gave written informed consent.

### Pathological evaluation

Therapeutic responses to preoperative CRT were evaluated with the surgically resected specimens. The excised tissues were fixed in buffered formalin and embedded in paraffin. In each case, the entire lesion was serially sliced at 4 mm for routine processing and embedding in paraffin. Then, 4-μm-thick sections were cut, stained with haematoxylin and eosin, and examined by light microscopy. Amounts of residual tumour mass, fibrotic changes, radiation vasculopathy, and peritumoural inflammatory reaction were checked, and therapeutic effects were assessed using Dworak grades (Dworak *et al*, 1997) as follows:

grade 0: no regression;
grade 1: dominant tumour mass with obvious fibrosis and/or vasculopathy;
grade 2: dominant fibrotic changes with few tumour cells or groups (easy to find);
grade 3: very few tumour cells in fibrotic tissue with or without mucous substance;
grade 4: no tumour cells, only fibrotic mass (total regression).

### Immunohistochemistry of Ki67, Mcm3, Bax, Bcl-2, ssDNA, Grp78, TS, DPD, CD34, VEGF, nestin, and LAT1

Formalin fixed, paraffin-embedded histological sections (4 μm in thickness) in tumour biopsies before CRT were immunostained for 12 antigens (Ki67, Mcm3, Bax, Bcl-2, ssDNA, Grp78, TS, DPD, CD34, VEGF, nestin, and LAT1). The antibodies used and methods for antigen retrieval are listed in Table 2. Endogenous peroxidase was blocked with 3.0% hydrogen peroxide for 10 min, and incubation with Protein block serum-free solution (DakoCytomation, Glostrup, Denmark) for 10 min. Sections were incubated with the anti-Ki67, Bax, Grp78, TS, DPD, and CD34 primary antibodies for 60 min at room temperature, and with the anti-Mcm3, Bcl-2, ssDNA, VEGF, and nestin antibodies overnight at 4°C. After incubation with either labelled polymer, anti-mouse, or anti-rabbit (EnVision+ System HPR; DakoCytomation) for 60 min at room temperature, 3,3′-diaminobenzidine was used as the chromogen. Nuclei were counterstained with methyl green solution to facilitate histopathological assessment. The immunohistochemical protocol with the LAT1 staining kit (Fuji Biomedix, Tokyo, Japan) was according to the manufacturer's manual.

### Evaluation of immunohistochemical staining

Ki67 and Mcm3 LI were determined as percentage values counting at least 1000 tumour cells in high-power fields ( × 400). With ssDNA staining, immunohistochemically positive cells were so few in number that at least 5000 nuclei were counted. ssDNA indices also were determined as percentage values.

Immunoreactivity for Bax, Bcl-2, Grp78, TS, DPD, and VEGF was evaluated using a score based on the classification of Sinicrope *et al* (1995). The staining intensity was scored as follows: none, 0; weak, 1; moderate, 2; intense, 3. If heterogeneity of staining intensity existed in a section, the staining intensity was scored based on that which was predominantly observed. The percentages of positive cells were assigned to one of five categories of protein expression: 0, ⩽5%; 1, 5–25%; 2, 25–50%; 3, 50–75%; 4, ⩾75%. The two scores were then multiplied to produce a weighted score for each tumour specimen. Two pathologists (MK and TM) independently scored the lesions and determined the final scores by discussion when they differed.

CD34-expressing capillaries were counted to give the MVD. Nestin-examined capillaries were considered as capillaries consisting of newly formed endothelial cells (Teranishi *et al*, 2007). Areas of highest neovascularisation were found by scanning tumour sections at low power ( × 100). The highest vascular counts of two different fields were averaged and used to calculate numbers of microvessels per mm^2^, defined as MVD with both stainings.

For LAT1, a biomarker for high-grade malignancy, staining intensity was scored according to a previous report (Sakata *et al*, 2009): none, 0; weak, 1; moderate, 2; intense, 3. The percentages of positive cells were assigned to one of four categories: 0, <0%; 1, 1–10%; 2, 10–30%; 3, >30%. The values for the two variables were then multiplied, resulting in a scoring from 0 to 9. Two pathologists (MK and TM) independently scored the lesions.

### Statistical analysis

Data were analysed using Dr. SPSS II (SPSS, Chicago, IL, USA) and Statview 5.0 (SAS Institute Inc., Cary, NC, USA) software. Immunohistochemical labelling and scores were compared using the Kruskal–Wallis test and the Mann–Whitney *U*-test. Logistic regression analysis was performed with a stepwise method. *P*<0.05 was considered as statistically significant.

## Results

### Pathological response to CRT

Pathological evaluation of responses to preoperative CRT in resected rectum revealed radiation effects in all cases, with fibrosis and vascular changes. All 60 cases were classified into Dworak regression grades 1–4. Of these, 15 (25.0%) showed complete pathological responses (regression grade 4) and 14 (23.3%) showed microscopic residual tumours (regression grade 3), whereas 21 (35.0%) and 10 (16.7%) showed moderate (regression grade 2) or minimal (regression grade 1) responses to preoperative CRT, respectively.

**Figure 1:**
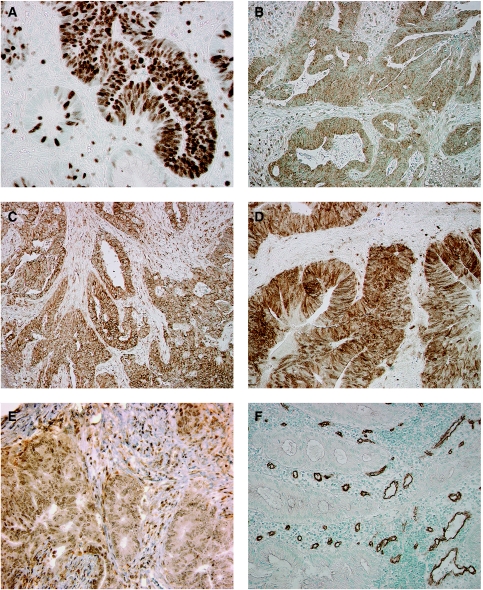
Immunohistochemical staining of pre-treatment rectal biopsy specimens from locally advanced rectal cancers. (**A**) Ki67 immunoreactivity, (**B**) Bax immunoreactivity, (**C**) Grp78 immunoreactivity, (**D**) TS immunoreactivity, (**E**) DPD immunoreactivity, and (**F**) CD34 immunoreactivity. Note Ki67 immunoreactivity confined to the tumour cell nuclei, Bax, and Grp78 to the tumour cell cytoplasm, TS and DPD to the tumour cell nucleus and cytoplasm, and CD34 to the endothelium of intratumoural microvessels.

Ki67, Mcm3, and ssDNA expression was confined to tumour cell nuclei, and Bax, Bcl-2, Grp78, and VEGF immunoreactivity to the tumour cell cytoplasm. TS and DPD were expressed in both nuclei and cytoplasm. LAT1 was confined to cell membranes, and CD34 and nestin were expressed in endothelium of intratumoural microvessels (Figure 1).

**Figure 2:**
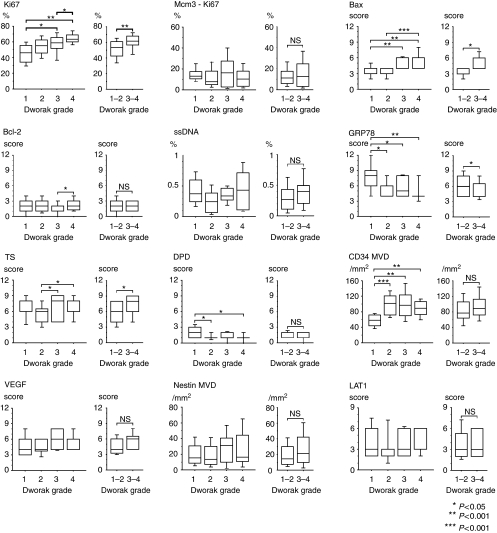
Ki67, Bax, Grp78, TS, DPD, and CD34 (MVD) were significantly related to chemoradiosensitivity (*P*<0.05). High Ki67 LI, Bax score, TS score, and low Grp78 were significantly correlated with tumour regression when responders were defined as having Dworak regression grades 3 and 4.

Figure 2 demonstrates the relationship between each immunohistochemical marker of the tumour biopsies before CRT and the pathological tumour response. A high Ki67 LI, Bax score, TS score, DPD score, MVD by CD34, and a low Grp78 score correlated with regression on univariate analysis. Recent studies have revealed that pathological CR and greater than 95% pathological response groups achieve a significantly improved overall survival and recurrence-free survival when compared with less than 95% pathological response groups (Ruo *et al*, 2002; Guillem *et al*, 2005). Therefore, we divided the cases into two groups: Dworak grades 1 and 2, and grades 3 and 4 (Gavioli *et al*, 2005). The latter were considered as responders to CRT. A high Ki67, Bax score, and TS score and a low Grp78 score were well correlated with response. On the other hand, there were no associations with the other immunohistochemical factors, as well as clinicopathological factors (Table 3).

### Multiple logistic regression analysis

Multiple logistic regression analysis was performed with a stepwise method (Tanaka *et al*, 1999). Independent variables were the data for Ki67 LI, Bax score, TS score, and Grp78 score, and dependent variables were no-response (0; Dworak regression grades 1 and 2) or response (1; Dworak regression grades 3 and 4). Other immunohistochemical markers and clinicopathological factors were not used. By the logistic regression analysis, we detected the Ki67 LI, Bax score, and TS score as independent factors (Table 4). The Bax score (odds ratio 18.1) had the strongest influence. The logistic regression formula was as follows: 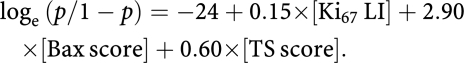


### Receiver-operating characteristic curve

**Figure 3:**
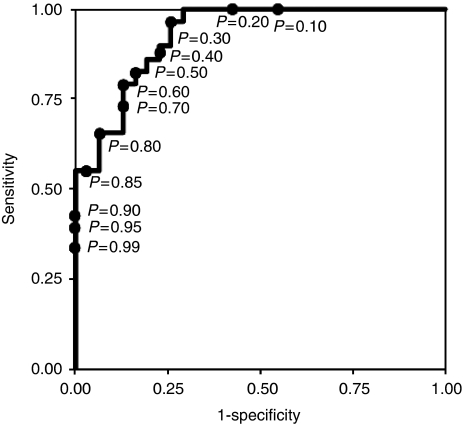
Receiver-operating characteristic curve with the logistic regression model. The area under the curve is 0.928 (95% confidence interval; 0.867–0.988).

A receiver-operating characteristic curve was generated by plotting the true-positive rate (sensitivity) on the *y* axis and the false-positive rate (1−specificity) on the *x* axis (Figure 3) (Tanaka *et al*, 1999).

Although the *P*-value at the point closest to the left upper corner on the curve is generally considered to represent the best balance of both sensitivity and specificity in distinguishing between response and no-response, we determined four points of *P* as the cut-off values (0.90, 0.50, 0.40, and 0.20) to construct practical criteria for the five categories ‘responder’, ‘probable responder’, ‘unknown’, ‘probable non-responder’, and ‘non-responder’ (Table 5). The points of *P*=0.90 and 0.20 meant the points of specificity 100% and sensitivity 100%, respectively. The point of *P*=0.50 meant the point at which the specificity was maximum and the sensitivity was more than 80%. The point of *P*=0.40 meant the point at which the specificity for prediction of non-responder was maximum and the sensitivity more than 80%.

### Sensitivity and specificity

A *P*-value for each case was calculated with three immunohistochemical markers examined in 60 sets of biopsy specimens. Using the calculated *P*-value, we classified the 60 patients into one of the above five categories with criteria distinguishing between responder and non-responder. Sensitivities and specificities of the criteria are shown in Table 5.

## Discussion

In this study, we sought clinicopathological factors and immunohistochemical markers that might contribute to prediction of chemoradiation effects on locally advanced rectal cancer. Our conclusion is that it is possible to predict a responder to preoperative CRT, with 82.8% sensitivity and 83.9% specificity, using the value calculated with the three elements of the Ki67 LI, the Bax score, and the TS score in biopsy specimens before CRT. In fact, high expression of Ki67, Bax, and TS was positively correlated with therapeutic effects.

The first factor, high proliferative activity with Ki67 as the marker, was earlier found to correlate with PCNA immunostaining, and mitotic counts after radiation of rectal cancer (Willett *et al*, 1995). Later, beneficial effects of radiotherapy for patients with various carcinoma with high Ki67 LIs were reported (Nakano *et al*, 1997; Raybaud-Diogene *et al*, 1997). However, in other reports, no relation was noted between Ki67 values in biopsy specimens before radiation and response rate in rectal cancers (Suzuki *et al*, 2004; Debucquoy *et al*, 2008). Suzuki *et al* (2004) performed preoperative radiotherapy only. Debucquoy *et al* (2008) combined preoperative radiotherapy and/or 5-FU/LV. Because we adopted CRT for all patients, the response may be more influenced by chemotherapy than radiation.

The second factor, Bax expression, was also reported by Chang *et al* (2005) to correlate well with chemoradiation therapeutic effects, and the authors considered that apoptosis may be important in rectal carcinoma response to CRT. Similarly, Bax overexpression has been found to correlate with anticancer drug sensitivity in a variety of human cancers, through enhanced induction of apoptosis (Krajewski *et al*, 1995; Guo *et al*, 2000; Teranishi *et al*, 2007). However, Gosens *et al* (2008) did not find any link between Bax expression and rectal cancer regression for neoadjuvant chemoradiation. They evaluated the regression grading system described by Rödel *et al* (2005): (1) no regression or <25% of tumour mass, (2) 25 to >50% tumour regression, and (3) complete regression. In addition, Bax immunohistochemical values were only intensity of cytoplasmic staining 0–3. Differences in grading systems and immunohistochemical expression scoring could clearly influence the results.

Rau *et al* (2003) immunohistochemically investigated the expression of p53, Bax, p21, Ki67, hMSH2 in pre- and post-therapeutic rectal carcinoma with preoperative radiotherapy. Only low p21 expression in tumour samples was significant in no-response to neoadjuvant chemoradiation. They reported no relation with Bax expression but classified responders as CR or partial response, histopathologically defined with resected post-therapeutic rectum, again differing from our definition as Dworak grades 3 or 4.

The third factor, TS, is important in pyrimidine nucleotide synthesis and represents an important chemotherapeutic target for 5-FU chemotherapy. Immunohistochemically, high TS expression in pre-treatment locally advanced rectal cancer biopsies was earlier shown to be predictive of a higher pathological response in the fluorouracil/oxaliplatin-base chemotherapy (Negri *et al*, 2008). A trend toward a direct correlation between the level of TS expression and response of 5-FU/LV treatment in patients with metastatic colon cancer has been noted (Johnston *et al*, 2003). Similar results have also been reported by Edler *et al* (2002) and Kornmann *et al* (2003).

However, low TS expression was a predictor of response to 5-FU chemotherapy for colorectal cancer metastases (Aschele *et al*, 1999) and advanced colorectal cancer (Cascinu *et al*, 1999). Aschele *et al* (1999) used a regimen of schedule-specific biochemical modulation of 5-FU plus methotrexate, and Cascinu *et al* (1999) applied 5-FU/LV. In both studies, cases with metastases and/or recurrence were included, and TS expression was evaluated as intensity 0 (undetectable staining) to 4 (very high intensity of staining), and then intensity levels 0–2 were considered as low, and 3 and 4 as high expression. We examined both cytoplasmic TS expression intensity and percentage of positive cells, as well as the Bax value. In another study, by Liersch *et al* (2006), TS expression was examined in surgically resected rectal cancer. In the reports, high TS expression correlated with cancer relapse. The clinical meaning of evaluation of TS expression needs further clarification.

The multiple logistic regression analysis revealed Ki67 LI, Bax score, and TS score to be independent factors, with a sensitivity and specificity for prediction of responder cases of 82.8 and 83.9%, respectively. Although the logarithm model is difficult to calculate for daily use, it can be easily converted to a linear model. It is sufficient for users to know the values of log_e_ (*P*/1−*P*) at the point of criteria. Practically, users can directly substitute the Ki67 LI, Bax score, and TS score into the formula: 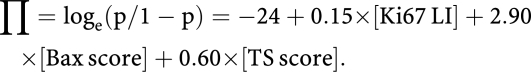


If this value Π (log_e_ (*P*/1−*P*)) is larger than 0.00, it indicates a responder case. If it is smaller than −0.41, it indicates a non-responder case (Table 5).

At present, CRT with subsequent surgical resection is performed without selection of cases. However, with our approach, likely responder cases can be chosen before therapy. In the future, our multivariate model should be revised using new factors to improve the sensitivity and specificity. The treatment strategy for locally advanced rectal cancer should be further developed toward so-called tailor-made therapy including such evaluation before preoperative therapy and/or surgical resection.

## Figures and Tables

**Table 1:** Characteristics of the patients

**Characteristic**	
Age (year) (mean±s.d.) (*n*=60)	63.9±10.6 (range 32–81)
Sex (male/female) (*n*=60)	44 (73.3%)/16 (26.7%)
Tumour size (mm) (mean±s.d.) (*n*=56)	47.4±17.2 (range 20–95)
	
*Clinical T stage (n=58)*	
cT3	56
cT4	2
	
*Clinical N stage (n=58)*	
cN0	32
cN1/cN2	26
	
*Histological type (biopsy) (n=60)*	
Well	36
Moderate	23
Poor	1
CEA (mg/100 ml) (mean±s.d.) (*n*=60)	8.9±12.7
CA19-9 (ng/ml) (mean±s.d.) (*n*=60)	19.7±27.2

s.d., standard deviation.

Normal ranges of CEA and CA19-9 were <5 mg/100 ml and <37 ng/ml, respectively.

Tumour size (based on double-contrast barium enema), clinical TN stage, and tumour markers were evaluated before chemoradiation therapy.

**Table 2:** Antibodies used for the immunohistochemical study

**Antibody**	**Clone**	**Source**	**Dilution**	**Antigen retrieval**
Ki67	Monoclonal MIB-1	DakoCytomation, Glostrup, Denmark	1 : 50	Treatment in hot bath (95–98°C) for 40 min (Dako Target Retrieval Solution (pH 9.0))
Mcm3	Monoclonal 3A2	MBL, Nagoya, Japan	1 : 400	Microwave treatment for 15 min (Dako Target Retrieval Solution (pH 9.0))
Bax	Monoclonal B-9	Santa Cruz Biotechnology, Inc., Santa Cruz, CA, USA	1 : 100	Microwave treatment for 15 min (citrate buffer (pH 6.0, 0.01 mol/l))
Bcl-2	Monoclonal 124	DakoCytomation	1 : 50	Microwave treatment for 15 min (Dako Target Retrieval Solution (pH 9.0))
ssDNA	Polyclonal A4506	DakoCytomation	1 : 400	Not applied
Grp78	Polyclonal H-129	Santa Cruz Biotechnology, Inc.	1 : 100	Microwave treatment for 30 min (citrate buffer (pH 6.0, 0.01 mol/l))
TS	Monoclonal TS106	DakoCytomation	1 : 50	Autoclave (121°C) treatment for 15 min (citrate buffer (pH 6.0, 0.01 mol/l))
DPD	Polyclonal RDPDPA	Taiho Pharmaceuticals Co., Ltd., Tokyo, Japan	1 : 400	Autoclave (121°C) treatment for 10 min (EDTA (pH 8.0, 1 mmol/l))
CD34	Monoclonal QBEnd 10	DakoCytomation	1 : 500	Not applied
VEGF	Polyclonal A-20	Santa Cruz Biotechnology, Inc.	1 : 100	Not applied
Nestin	Polyclonal N1602	IBL, Takasaki, Japan	1 : 500	Not applied
LAT1	Monoclonal	Fuji Biomedix, Tokyo, Japan	Prediluted	Microwave treatment for 5 min (in the buffer supplied by kit)

Mcm, minichromosome maintenance; Grp78, glucose-regulated protein 78; TS, thymidylate synthase; DPD, dihydropyrimidine dehydrogenase; VEGF, vascular endothelial growth factor; LAT1, L-type amino-acid transporter 1.

**Table 3:** Clinicopathological characteristics of the patients separated by Dworak grades 1, 2 *vs* 3, 4

	**Dworak grade 3, 4 (responder) (*n*=29)**	**Dworak grade 1, 2 (non-responder) (*n*=31)**	
Age (year) (mean±s.d.)	63.5±11.4	63.5±9.8	*P*=0.11
*Sex*			
Male	21	24	
Female	8	7	*P*=0.65
Tumor size (mm) (mean±s.d.)	46.7±14.4	48.0±19.7	*P*=0.98
			
*Histological type (biopsy)*			
Well	17	20	
mod/por	12	11	*P*=0.64
CEA (mg/100 ml) (mean±s.d.)	8.5±12.7	9.4±8.5	*P*=0.23
CA19-9 (ng/ml) (mean±s.d.)	17±25	22±29	*P*=0.054

Well, well-differentiated adenocarcinoma; mod/por, moderately to poorly differentiated adenocarcinoma; s.d., standard deviation.

**Table 4:** Results of multiple logistic regression analysis

	**Regression coefficient**	***P*-value**	**Odds ratio**	**95% CI**
*Variable*				
Ki67 LI	0.15	0.002	1.17	1.06–1.29
Bax score	2.90	0.001	18.1	3.11–105.7
TS score	0.60	0.019	1.83	1.11–3.03
Constant	−24.47	<0.001		

LI, labelling index; CI, confidence interval.

**Table 5:** Criteria for Dworak grades 1, 2 *vs* 3, 4, and their validities tested among the 60 patients

			**Pathological response**		**Validity**			
			**DG 3, 4**	**DG1, 2**	**DG 3, 4**		**DG 1, 2**	
**Category**	**Definition (*P*)**	**Definition (Π)**	**Responder (*n*=29)**	**Non-responder (*n*=31)**	**Se**	**Sp**	**Se**	**Sp**
Responder	0.90⩽*P*	2.20⩽Π	13	0	44.8	100		
Probable responder	0.50⩽*P*<0.90	0⩽Π<2.20	11	5	82.8	83.9		
Unknown	0.40⩽*P*<0.50	−0.41<Π<0	1	1				
Probable no-responder	0.20<*P*⩽0.40	−1.39<Π⩽−0.41	4	7			80.6	86.2
No-responder	*P*⩽0.20	Π⩽−1.39	0	18			58.1	100

*P*, probability; DG, Dworak grade; Se, sensitivity; Sp, specificity; Π=log_e_ (*P*/1−*P*).
